# Effect of ecological and anthropogenic factors on grouping patterns in African lions across Kenya

**DOI:** 10.1002/ece3.10982

**Published:** 2024-02-14

**Authors:** Mumbi Chege, Laura D. Bertola, Geert R. De Snoo, Shadrack Ngene, Tobias Otieno, Irene Amoke, Maarten van 't Zelfde, Stephanie Dolrenry, Femke Broekhuis, Will Tamis, Hans H. De Iongh, Nicholas B. Elliot

**Affiliations:** ^1^ Wildlife Research and Training Institute Naivasha Kenya; ^2^ Institute of Environmental Sciences CML Leiden University Leiden The Netherlands; ^3^ Department of Biology University of Copenhagen Copenhagen Denmark; ^4^ Netherlands Institute of Ecology (NIOO‐KNAW) Wageningen The Netherlands; ^5^ Ewaso Lions Nairobi Kenya; ^6^ Kenya Wildlife Trust Nairobi Kenya; ^7^ Lion Guardians Nairobi Kenya; ^8^ Wildlife Ecology and Conservation Group Wageningen University and Research Wageningen The Netherlands; ^9^ Department of Evolutionary Ecology, Department Biology University of Antwerp Antwerpen Belgium; ^10^ Wildlife Counts Nairobi Kenya

**Keywords:** African lion, behavior, fission‐fusion, group size, Kenya, sociality

## Abstract

Social carnivores frequently live in fission–fusion societies, where individuals that share a common territory or home range may be found alone, in subgroups, or altogether. Absolute group size and subgroup size is expected to vary according to resource distribution, but for species that are susceptible to anthropogenic pressures, other factors may be important drivers. African lions (*Panthera leo*) are the only truly social felid and lion prides are characterized by fission–fusion dynamics with social groups frequently splitting and reforming, and subgroup membership can change continuously and frequently. The number of individuals in a group can be reflective of social, ecological, and anthropogenic conditions. This dynamic behavior makes understanding lion grouping patterns crucial for tailoring conservation measures. The evolution of group living in lions has been the topic of numerous studies, and we drew on these to formulate hypotheses relating to group size and subgroup size variation. Based on data collected from 199 lion groups across eight sites in Kenya, we found that group sizes were smaller when lions were closer to human settlements, suggesting that edge effects are impacting lions at a national scale. Smaller groups were also more likely when they were far from water, and were associated with very low and very high levels of non‐tree vegetation. We found significant differences between the study sites, with the Maasai Mara having the largest groups (mean ± SD = 7.7 ± 4.7, range = 1–19), and Amboseli conservation area the smallest (4.3 ± 3.5, range = 1–14). While long‐term studies within a single site are well suited to thoroughly differentiate between absolute group size and subgroup size, our study provides unique insight into the correlates of grouping patterns in a vulnerable species at a national scale.

## Graphical abstract

Social carnivores, such as lions, commonly reside in fission–fusion societies, where individuals may be found alone, in subgroups, or together within a shared territory. While the impact of ecological and anthropogenic conditions on grouping patterns is known at broader scales, the effects of fine‐scale variations remain less explored. Lions, as the only truly social felid, exhibit fission–fusion dynamics with frequent and continuous changes in subgroup membership. Through data collected from eight sites across Kenya, we looked at the intricate relationship between ecological and anthropogenic factors and the grouping patterns of lions and observed distinct variations in both group and subgroup sizes at local scales.

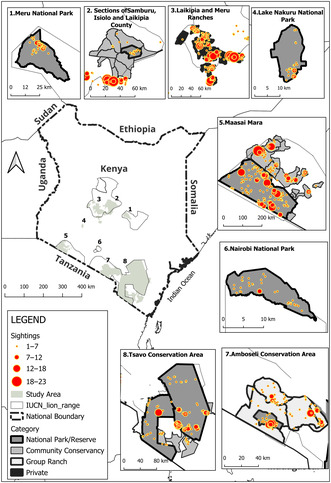

## 1 INTRODUCTION

Group living is a common behavior in the animal kingdom and may have evolved independently in various taxa (Majolo & Huang, 2018; Packer et al., 1990). However, many species remain solitary or exhibit social behavior only seasonally (Gager et al., 2016). The decision to live in groups or alone is influenced by environmental and social factors including, but not limited to, resource availability, access to mating opportunities and predation pressure (Krause & Ruxton, 2002; Majolo & Huang, 2018). These factors vary over time and space, and depending on local environmental conditions, groups are expected to have an optimal range of size. If a group falls below the lower limit of its optimal range, individuals may choose to join another group. Conversely, if it exceeds the upper limit of its optimal range, the costs may outweigh the benefits and individuals may choose to split and form new smaller groups to enhance their fitness and survival chance (Packer et al., 1990; Valeix et al., 2012). The drivers of optimal group size vary among species and habitats. For example, studies have shown that intermediate‐sized groups of baboons (*Papio cynocephalus*) have more efficient space‐use strategies than larger or smaller groups (Markham et al., 2015). In social carnivores such as African wild dogs (*Lycaon pictus*) and African lions (*Panthera leo*), optimal group size is thought to be determined by factors relating to foraging, breeding, and survival (Courchamp et al., 2002; Packer et al., 1990; VanderWaal et al., 2009).

The spatial distribution and grouping patterns of female mammals has a significant impact on male intrasexual competition, and is thought to be pivotal in driving social evolution (Majolo & Huang, 2018). The social dynamics of lions are characterized by the formation of groups (prides), which are relatively stable social units, consisting of related females and their offspring (Schaller, 1972). Prides grow in number through the recruitment of daughters, but an increased proportion of daughters will remain or disperse from the natal pride depending on whether potential pride size exceeds the habitat‐specific optimum (VanderWaal et al., 2009). Prides are characterized by fission–fusion dynamics, and temporarily break up into sub‐units referred to as subgroups (Mosser & Packer, 2009; Schaller, 1972). Lion grouping patterns are influenced by social and environmental conditions that lead to continuous and constant changes (Mosser & Packer, 2009). For example, Mbizah et al. (2020) found that resource availability and dispersion play a crucial role in individual decisions concerning associations; where the optimal group size for lions was dependent on prey availability, with solitary lions preying on small prey, large groups preying on larger prey, and smaller to medium sub‐groups forming when prey was abundant. Mosser et al. (2009) found that proximity to river confluences was the best predictor of female reproductive success, and that larger prides were better able to acquire and keep control of the best quality habitats. Group size is also important for male coalitions, since larger coalitions are more likely to obtain residency and therefore have greater reproductive success (Borrego et al., 2018).

Anthropogenic factors can disrupt grouping patterns in large carnivores that occur in close proximity to people. Livestock depredation is common where carnivores and humans co‐occur, which often leads to the retaliatory killing of carnivores (Dickman et al., 2011; Harcourt et al., 2001). Such conflicts represent a major source of mortality for carnivores and wildlife area boundaries are frequently population sinks (Woodroffe & Ginsberg, 1998). These so called “edge effects” can alter group size either by direct killing of group members, or because groups fission into subgroups to avoid detection. For instance, in Hwange National Park (NP), pride size was smaller close to the park boundary, since lions there suffered direct persecution (Loveridge et al., 2010). However, in the group ranches around Amboseli NP, Dolrenry (2013) observed that after being chased by a “Maasai hunting party” lion groups of up to 10 individuals separated into pairs or singles for days or several years, probably to avoid detection by humans.

**FIGURE 1:**
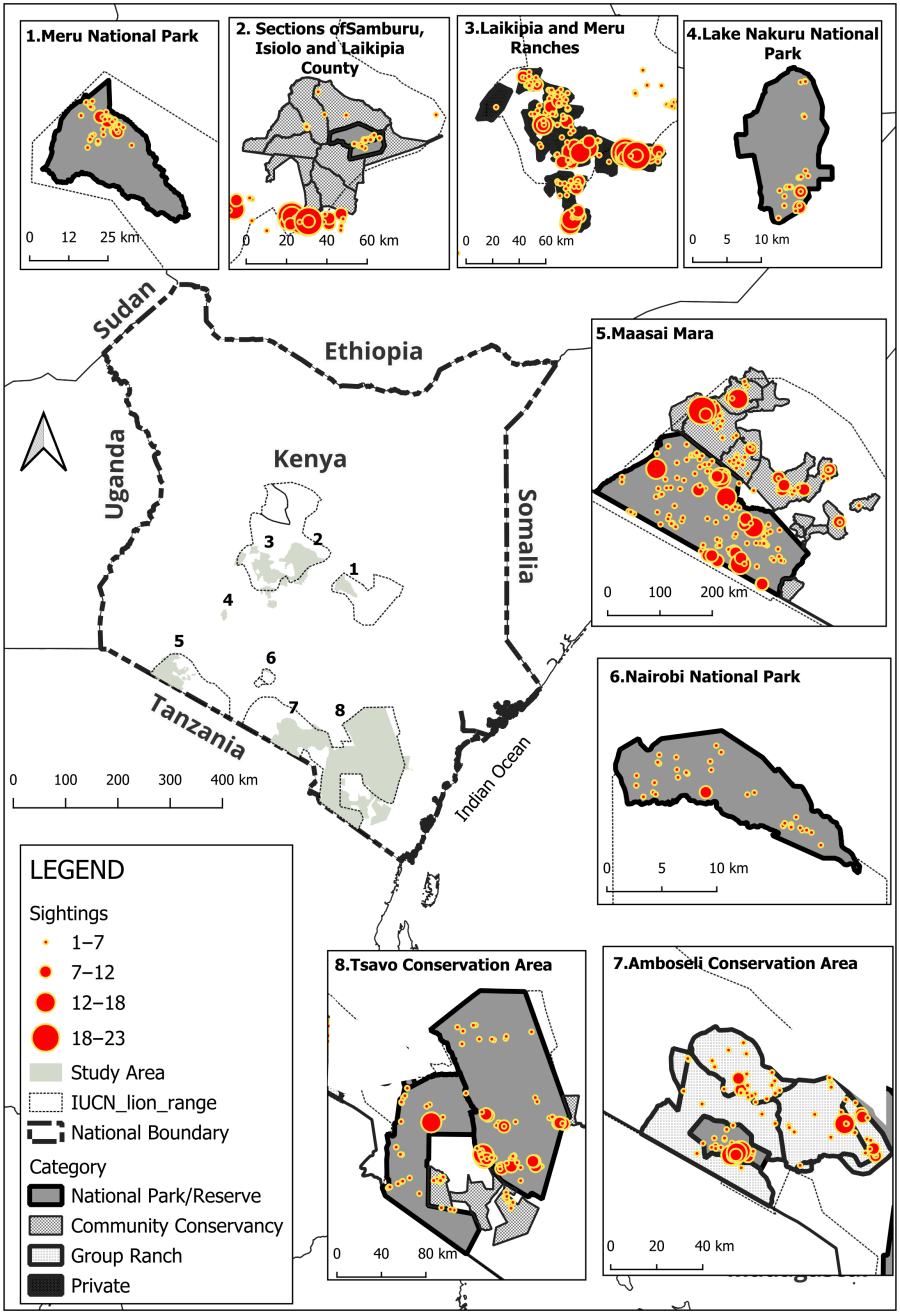
Location of the study sites in Kenya. For additional description of the individual study areas please see Table 1.

Group living is a vital aspect of lion adaptation and persistence, and it is, therefore, necessary to understand how ecological and anthropogenic factors influence lion grouping patterns. This understanding is important as it sheds light on how local conditions impact not only grouping patterns but also the broader implications on lion populations and the strategies required for their management. We, therefore, explored the influence of land management and a range of ecological and anthropogenic variables on lion group size on a national scale in Kenya. To our knowledge, this is the first study to look at lion grouping patterns on a large spatial scale across a variety of land management types. Lions in the country are distributed across a fragmented network of government protected areas, community conservancies, group ranches, and private conservation areas that differ in land ownership and, therefore, in management. We covered eight study sites in Kenya known to host resident lion populations (Figure 1). We defined the following research questions, with associated hypotheses in Table 1:

1. Does lion group size vary by (a) land management or designation type and (b) per study site irrespective of land management type?
2. How do ecological and anthropogenic factors affect lion subgroup size within the different study sites?

**TABLE 1:** Description of ecological and anthropogenic variables used to determine factors influencing lion group size in Kenya.

Land management type: We hypothesize that National Parks and Reserves will have larger lion group sizes compared to the other land management types since land that is under an official wildlife protection status is associated with higher protection from human activities (John Waweru et al., 2021)				
**Variables**	**Code**	**Variable description**	**Hypothesis**	**Citation(s)**
Ecological				
Habitat	Non‐tree	Data were derived from Modis Vegetation Continuous Fields data. A primary data layer which describes the percent of each pixel covered by non‐tree vegetation canopy (grass, bush and shrub lands). Available in .tiff format, the dataset was resampled to 1 km^2^ resolution.	Larger subgroups will be in areas with moderate proportion non tree cover	Mosser et al. (2009) and Mudumba et al. (2015)
Water availability (Distance to rivers (m))	Dist_riv	Euclidean distance tool in Arcmap was used to measure distance (m) from each drainage line at 1 km^2^ resolution.	Larger subgroups will be found close water	Hopcraft et al. (2005) and Mosser et al. (2009, 2015)
Anthropogenic				
Distance to human settlements (m)	Dist_hs	NEAR tool was used to measure distance (m) from each lion sighting to the nearest human settlement.	Large subgroups will be found further away from human settlements	Dolrenry (2013), Oriol‐Cotterill et al. (2015), Valeix et al. (2012) and Woodroffe (2000)
Distance to boundary (m)	Dist_bound	NEAR tool was used to measure distance (m) from each lion sighting to the area boundary	Large subgroups will be further away from boundaries	Dolrenry (2013), Oriol‐Cotterill et al. (2015) and Valeix et al. (2012)

## 2 METHODS

### 2.1 Study area

Kenya's land area covers ~582,646 km^2^, of which approximately 2% is covered by water (IGAD, 2010). It has a tropical savannah climate that is characterized by an average annual temperature ranging from 10 to 26°C. The annual rainfall increases from the northeast to the southwest and ranges from 250 to 2000 mm (Zhou et al., 2017). The pattern is bimodal, with long rains from March to June and short rains from October to December (wet season), the rest of the months constitute the dry season. Rangelands, which are cultivated lands that are primarily used for grazing and browsing of wildlife and livestock, make up over 80% of Kenya's land mass and are characterized by an arid and semi‐arid climate (Denboba, 2022). They are essential for wildlife conservation and livestock farming and by 2009, they hosted 70% of the country's livestock and a population of 12 million people (Ogutu et al., 2016). About 8% of Kenya's land area is under wildlife conservation by the state and includes a system of National Parks and National Reserves (The Wildlife Conservation and Management ACT, 2013). An additional 11% consists of community‐owned conservancies and group ranches and private ranches/conservancies owned and managed by individuals, elected officials, or corporate bodies (Kenya Wildlife Conservancies Association, 2019).

Kenya's lion populations face a myriad of threats such as conflicts with local communities, habitat loss, loss of wild prey (Ogutu et al., 2016), and disease (Kenya Wildlife Service, 2016). Despite these threats, lions are predicted to be widely distributed across the country (Broekhuis et al., 2022), with the largest population found in the Maasai Mara, followed by Tsavo, Laikipia, and Amboseli, with several other populations of less than 100 individuals (Elliot et al., 2021).

### 2.2 Data collection and acquisition

All data were collected between 2017 and 2019 primarily during the dry seasons, during a national survey, aimed at estimating lion density within potential source populations. Field methods are detailed in Elliot et al. (2021), but briefly, unstructured spatial sampling protocols were deployed to collect data within a spatial capture–recapture framework. A primary objective of the fieldwork was to individually identify as many lions as possible, as many times as possible, while uniformly covering the survey area. During each survey, when lions were sighted, a series of close‐up photographs were taken of each individual from different angles so as to acquire records of their unique vibrissae spots (Pennycuick & Rudnai, 1970). The location of all individuals was also recorded, and when more than one lion was seen at a single sighting, the number of all lions observed together was recorded. This included adults, sub‐adults, juveniles, and cubs. Each individual was then assigned a unique ID and gender based on secondary sexual characteristics and age based on phenotypic features, that is, body size, shoulder height, nose pigmentation, and mane development (Miller et al., 2016). Each individual was then assigned group membership (groupID) based on the field observations of the social groupings of individual lions. For example, if four lions were sighted together and identified, each would be given a unique ID and then all four would be assigned to the same group. In subsequent sightings, if new lions were seen with these identified individuals, they were then considered to be part of the same group.

Data were collected in the following eight sites, which are hereafter referred to by the name in brackets: (1) Meru conservation area (Meru), (2) Laikipia and Meru Ranches (Laikipia), (3) Sections of Samburu, Isiolo and Laikipia Counties (Samburu), (4) Lake Nakuru National Park (Nakuru), (5) Maasai Mara (Mara), (6) Nairobi National Park (Nairobi), (7) Amboseli conservation area (Amboseli), and (8) Tsavo conservation area (Tsavo) (Figure 1, Table 2). These areas cover National Parks and National Reserves, Private and Community Conservancies, Group Ranches, and buffer zones, as described in Elliot et al. (2021). Four sites have multiple management, for example, Mara (National reserve and community conservancies), Tsavo (community conservancies and national parks), Samburu (National reserve and community conservancies), and Amboseli (group ranches and national park). The length of each survey differed by site (mean number of days: 77, range = 22–105).

**TABLE 2:** Study area characteristics.

	Study site	Land management type	Year surveyed	Season when survey was conducted	Annual precipitation (mm)	Vegetation	Human pressure
1	Meru conservation area (Meru)	National Park	September to December 2019	Dry/Wet	200–724	Semi‐arid to very arid area characterized by *Combretum* and *Terminalia* wooded grasslands, and *Acacia* woodlands	Agriculture west of the conservation area, pastoralism to the east, south, and north (Elliot et al., 2021) Illegal livestock incursions (John Waweru et al., 2021)
2	Laikipia and Meru Ranches (Laikipia)	Private Conservancies (A total of 31 privately owned conservancies)	August to November 2019	Dry	400–1000	Semi‐arid to humid characterized by *V. drepanolobium* woodlands, with savannahs dominated by perennial grasses and intermittent trees and shrubs and bushlands (Elliot et al., 2021)	Land use includes commercial ranching, wildlife tourism, and small‐scale agriculture (Elliot et al., 2021)
3	Sections of Samburu, Isiolo, and Laikipia Counties (Samburu)	2 National Reserves and 9 Community Conservancies	August to November 2019	Dry	<400	Semi‐arid area characterized by scrub woodland and wooded grassland. Along the rivers—doum palm and acacia trees (Bhalla, 2017)	Increasing human settlements, illegal influx of livestock, and insecurity (Bhalla, 2017; John Waweru et al., 2021)
4	Lake Nakuru National Park (Nakuru)	National Park	September to October 2017	Dry	869	Characterized by vegetation characteristic of saline water ecosystems as well as grassland, scrub woodland, acacia woodland (Thuo et al., 2015)	Bordered by Nakuru city (north), intensive agricultural lands (west and south), and to the east by Soysambu Conservancy (Elliot et al., 2021)
5	Maasai Mara (Mara)	Maasai Mara National Reserve and 11 Community Conservancies	August to October 2018	Dry	650–1300	Open grasslands interspersed with woodlands and shrub lands (Elliot & Gopalaswamy, 2017)	Intensive agricultural land to the west and pastoralist settlement to the east (Elliot et al., 2021)
6	Nairobi National Park (Nairobi)	National Park	October to November 2018	Dry/Wet	524–912	Characterized by open grasslands, wooded riverine vegetation, shrubland, bushland, and forest (Lesilau et al., 2021)	Bordered by human settlements on the northern, eastern, and western borders (Elliot et al., 2021)
7	Amboseli Conservation Area (Amboseli)	Amboseli National Park and 3 Group Ranches	August to November 2018	Dry	200–500	*Acacia tortilis* woodland, mixed *Acacia mellifera*, *Commiphora rostrata* bushlands, and open grasslands (Okello et al., 2015) Has two perennial rivers and the swamps of the Amboseli basin provide a permanent water source (Dolrenry, 2013)	Land subdivision and subsequent conversion to agriculture and other uses that exclude wildlife, unplanned expansion of human settlements (Kenya Wildlife Service, 2020)
8	Tsavo Conservation Area (Tsavo)	3 National Parks and 17 Community Conservancies	January to April 2019	Dry	200–700	Semi‐arid area characterized by wooded bushland, shrub land and savannah grassland, and montane forests at the high elevation areas (Elliot et al., 2021)	Mining activities, illegal livestock grazing, increasing human population along park and conservancy boundaries increase in public infrastructure, that is, road and railway Cattle ranching and agriculture (Elliot et al., 2021; Mukeka et al., 2020)

### 2.3 Data management and analysis

#### 2.3.1 Lion group size

Lion group size was expressed as the number of lions (including adults, sub‐adults, juveniles, and cubs—excluding single adult males and coalitions) observed together per sighting, adapted according to Smuts et al. (1978). Sightings that consisted purely of unidentified individual(s) were removed (705 sightings) from the analysis since their group membership could not be assigned. Although we did not have long‐term data to differentiate with certainty the divergence between absolute group size and subgroup size, we considered two aspects of group membership, consistent with (Mbizah et al., 2020): (1) group size—the maximum number of individuals seen within a group at any one time, (2) subgroup size—the number of individuals present at each observation. To explore large‐scale differences across land management type and between sites we used group size, whereas to explore the influence of variables at a finer scale and on fission–fusion dynamics, we used subgroup size.

#### 2.3.2 Land management type

Each of the areas surveyed (Figure 1) was classified according to the existing legal management system as described below:

1. Community Conservancy (CC): land is owned by communities and managed by a management company. In this type of management, community members, livestock, and wildlife may share the land and there are systems in place to regulate resource use.
2. Group Ranches (GR): Land that is owned and used equally by group members, established primarily for livestock grazing. Wildlife may be present on the lands but with limited to no active management of wildlife.
3. National Parks and National Reserves (NPR): Wildlife conservation areas owned by either the national or county government, with a mandate for wildlife conservation.
4. Private Conservancies (PC): owned and managed by a private individual or corporate body, and generally practice integrated wildlife and livestock management.

### 2.4 Data preparation: explanatory variables

Since each lion observation was associated with a precise spatial location, and we were interested in understanding how subgroup size might relate to a set of spatial variables, for each lion observation we created a 1‐km buffer around the point and then extracted the mean pixel value for the following layers:

#### 2.4.1 Habitat

##### Non‐tree vegetation

Lions mostly occur in habitats that provide adequate cover for hunting such as grasslands, shrublands, riverine areas, and bushlands; but typically avoid very open and densely vegetated habitats (Lesilau et al., 2021; Mudumba et al., 2015; Spong, 2002). We hypothesized a quadratic relationship with larger groups being associated with medium proportions of non‐tree cover. We downloaded non‐tree vegetation data from USGS Modis continuous fields data (https://earthexplorer.usgs.gov) at 250 m spatial resolution for the year 2019. This non‐tree vegetation layer describes the percent of each pixel covered by non‐tree vegetation canopy (i.e., grass and shrubland). The values range from 0% to 100%, where 100% signifies 100% non‐tree cover/bush cover. The data were then resampled to 1 km^2^.

#### 2.4.2 Water availability

##### Rivers

Riverine areas generally represent high quality habitats since they provide shelter for cubs, and opportunity to ambush water‐dependent prey. Larger lion groups are better able to defend and maintain such areas (Mosser et al., 2009, 2015). We, therefore, expected larger groups in close proximity to rivers. River data were downloaded from the Digital chart of the world (http://diva‐gis.org/gdata). Large water bodies were digitized on Google Earth and the polygons were converted to polylines and then merged with the rest of the dataset. We then calculated the Euclidean distance to each polyline and resampled the resulting raster at 1 km^2^.

#### 2.4.3 Anthropogenic factors

##### Distance to human settlements

We downloaded GRID3 Republic of Kenya Settlement Extents Version 01.01 data, which is a derivative work from Digitize Africa. This dataset represents human settlements as polygons, with the boundaries of these settlements defined using building footprint and the year 2020 human population data. We then selected settlements that had a population density of above 25 people/km^2^ (population UN adjusted) based on Woodroffe (2000), who suggested that when human density exceeds 25 people/km^2^, lions become extirpated. We then calculated the distance from each lion observation to the human settlement polygons.

##### Distance to a boundary

We dissolved the internal administrative boundaries of adjoining areas and only considered the outer boundary of each site. For example, for the Mara we used the outer boundaries of the National Reserve and the Community Conservancies (Figure 1). We then calculated the distance from each lion sighting to the outer boundary of the conservation areas.

### 2.5 Data analysis

Data analysis was carried out on two levels: at a broad scale we assessed the mean of the maximum number of groups observed per land designation type and per study site, and at a finer scale, we explored the influence of variables on subgroup size. All statistical tests were carried out in RStudio using R3.4.4 (R Core Team, 2018).

To test for differences in lion group sizes per land management type and within each study site, we used a non‐parametric Kruskal–Wallis test. A Mann–Whitney *U* test was used to test for differences in lion group size in the four sites with multiple land management. We then tested the linear and quadratic (using the both linear and quadratic terms of each variable) relationship between the ecological variables and lion subgroup size for each study site using simple linear models. From these simple linear models, we considered the relationship between lion subgroup size and a variable to be quadratic if the output was significant, that is, the *p* value of the model with the quadratic term fell within .001–.1. We then developed a full generalized linear mixed model, that is, Poisson model (−1), that contained all the variables either in quadratic or non‐quadratic form, then following the step‐down model building approach, terms with high *p* values were manually removed through backwards stepwise model simplification obeying the principle of marginality (Kuznetsova et al., 2017). We accounted for repeat lion observations by taking groupID as a random factor, and for models that reported over‐dispersion, a random term, that is, (1|ID) was added to the mixed model to correct for over‐dispersion. This process was followed for all eight study sites.

## 3 RESULTS

**FIGURE 2:**
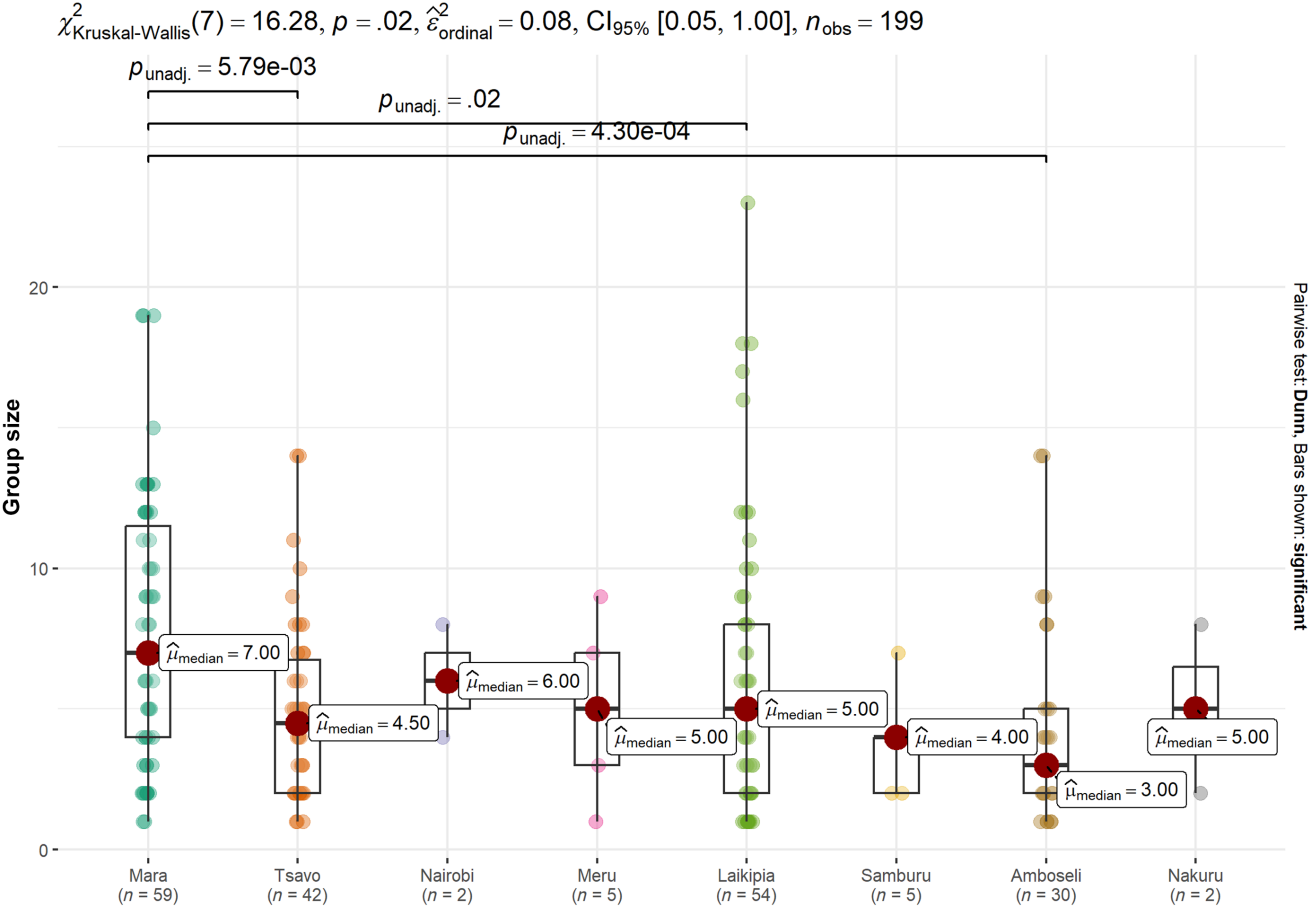
Mean lion group size per study site. The lines on the top show the significant pairwise comparisons, dots represent the sightings, boxes represent the interquartile range, circles indicate the median, and whiskers indicate 10th and 90th percentiles, the colors of the boxes correspond to the study sites.

Across all eight sites, we recorded a total of 1088 sightings of lions, which amounted to 3542 detections of individual lions (including single adult males and coalitions), many of which were seen multiple times. Based on our observations of lion associations, we documented 199 groups (excluding single adult males and those in coalitions), with the number of groups recorded in each site varying considerably (range 2–59, Figure 2).

### 3.1 Between‐study site group size

Across all sites, sub‐group size varied according to ecological and anthropogenic variables. Lion subgroup size increased further away from settlements, and decreased further away from rivers and the nearest boundary. We found a quadratic relationship between subgroup size and non‐tree vegetation with smaller subgroups being associated with very low and high levels of non‐tree vegetation (Table 3).

**TABLE 3:** Summary of the relationship between environmental and anthropogenic variables and lion group size per study site. Significant codes are indicated below the table.

Conservation area	Habitat	Water availability	Anthropogenic factors	
	Non‐tree	River distance	Distance from human settlements	Distance to boundary
All sites	o−***	−*	+*	−*
Meru		−^.^		−*
Laikipia		−*	+**	−^.^
Samburu	+*	−**	−^.^	
Nakuru		−*	+***	−***
Mara		−*		−**
Nairobi		+^.^		
Amboseli	o−*			
Tsavo	o−*			

*Note*: + indicates a significant positive relationship with lion subgroup size, − indicates a significant negative relationship with lion subgroup size, o indicated quadratic relationship between variable and lion subgroup size.

***p < .001; **p < .01; *p < .05 and ^.^ *p* < .1.

**FIGURE 3:**
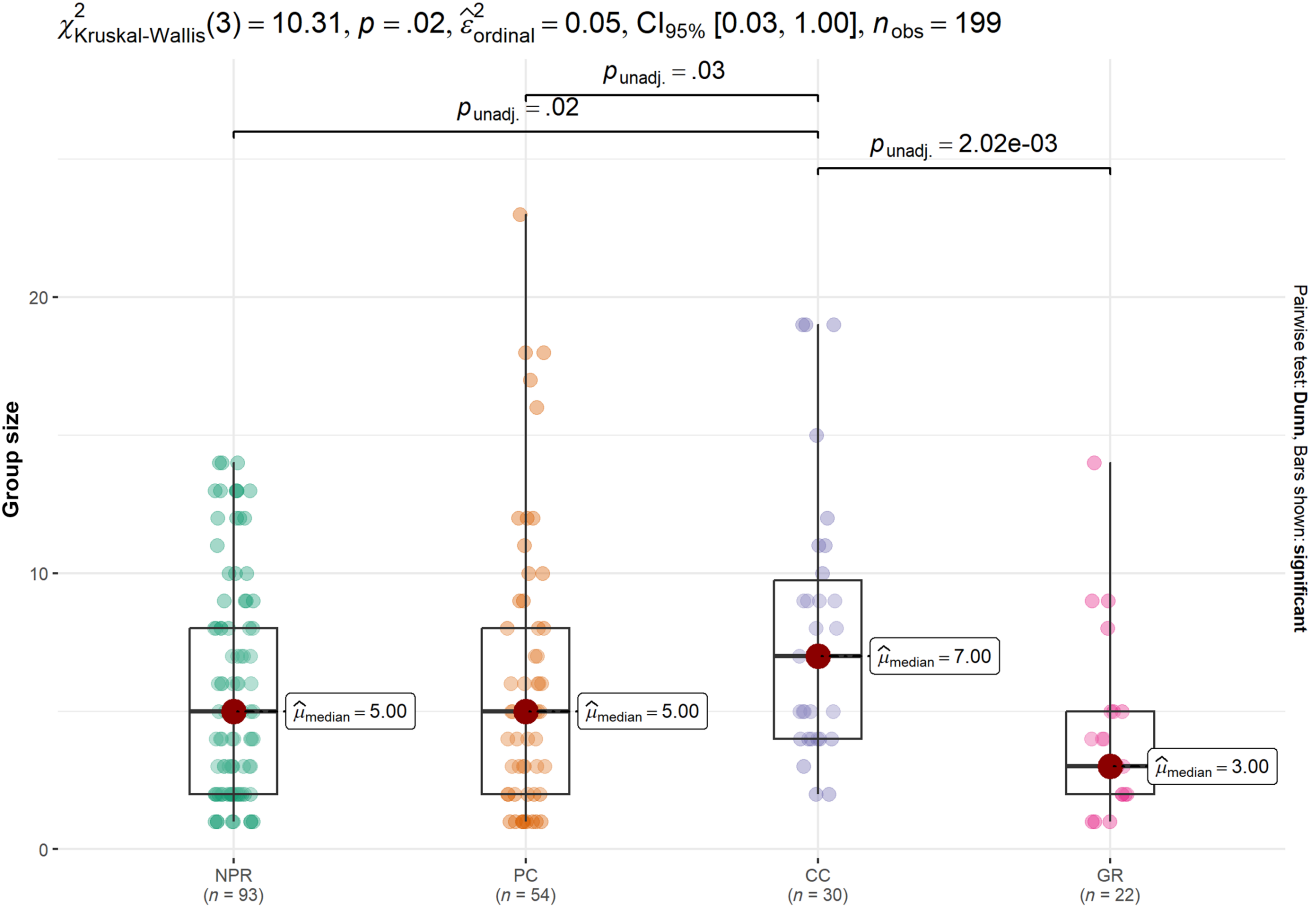
Mean lion group size per land management/designation. The lines on the top show the significant, pairwise comparisons, dots represent the sightings, boxes represent the interquartile range, circles indicate, the median and whiskers indicate 10th and 90th percentiles, the colors of the boxes correspond to the land management type.

The Kruskal–Wallis test indicated significant differences between land management types (*χ* ^2^(2) = 10.3, df = 3, *p*‐value = .016) (Figure 3). Community Conservancies had the largest group sizes (mean ± SD = 7.9 ± 4.9, range = 2–19), followed by Private Conservancies (6.1 ± 5.1, range = 1–23), National Parks and Reserves (5.7 ± 3.9, range = 1–14), and Group Ranches (4.2 ± 3.2, range = 1–14). We also found significant differences between the study sites (*χ* ^2^(2) = 16.3, df = 7, *p*‐value = .023), with the Mara having larger groups (mean ± SD = 7.7 ± 4.7, range = 1–19) than Tsavo (4.9 ± 3.3 range = 1–14), Laikipia (6.1 ± 5.1, range = 1–23) and Amboseli (4.3 ± 3.5, range = 1–14) (Figure 2). Within sites that had multiple management types, only the Mara showed significant differences, with Community Conservancies having larger groups (9.6 ± 4.9, range = 3–19) than the National Reserve (6.6 ± 4.3, range = 1–13) (Mann–Whitney *U* = 526, *p*‐value = .04).

### 3.2 Within‐ study site subgroup variation

For the individual sites, distance to river appeared most often, with negative relationships found for Laikipia, Mara, Meru, Nakuru, and Samburu. In other words, subgroup size decreased further away from rivers. The proportion of non‐tree vegetation had a quadratic relationship with subgroup size in Tsavo and Amboseli, where larger groups were most associated with intermediate proportions of non‐tree vegetation. In Samburu, lion subgroup size increased with increased non‐tree vegetation cover. In Laikipia and Nakuru, subgroup size increased further away from human settlements but decreased with distance from human settlements in Samburu. Subgroup size had a negative relationship with wildlife boundaries in the Mara and Nakuru, as groups were larger closer to boundaries. It is worth noting that in some models (where there were few observations) the marginal and conditional *R* ^2^ measures were notably low. Table 3 gives a summary of the results of the full models showing which variable(s) significantly contributed to the model for each site, as well as the relationship of the variable(s) with lion subgroup size (i.e., positive or negative). While Tables A1 and A2 (in the Appendix 1) give a summary of the output of the models, showing the significant variables for each study site.

## 4 DISCUSSION

We found that subgroup sizes were smaller close to human settlements. While this is not surprising given that lions may be persecuted when they overlap with humans, our results suggest that at a national level, lions may be subjected to edge effects (Woodroffe & Ginsberg, 1998). Although the term “edge effects” typically refers to areas close to wildlife area boundaries, our results reveal a nuance that a boundary in and of itself is not likely to limit lion populations. Indeed, our finding was opposite to what we had predicted, larger subgroups were found closer to boundaries. Since the boundaries themselves do not necessarily translate to anthropogenic impact, we speculate that other factors, such as habitat and prey are likely to be important (Mbizah et al., 2020; Mosser & Packer, 2009). We also found that lion subgroup sizes were smaller further away from water. This is in line with our expectations, since riverine habitats represent quality habitats that larger groups are better able to defend. In arid ecosystems, water sources also represent areas of more abundant prey, that can support larger groups.

Contrary to our hypothesis, we found that Community Conservancies rather than National Parks and Reserves, had larger group sizes. A similar pattern was also observed for the Mara when we examined differences among study sites with multi‐land management. We attribute the larger group sizes in CC to: (1) the period within which our data was collected may have coincided with a time when lions were driven to form large groups either due to presence of cubs or distribution of resources or (2) to the presence of a larger lion population. Indeed, Elliot and Gopalaswamy (2017) found that lion density in the Mara conservancies was higher than in the National Reserve. Another possibility could be related to the overall pride sizes and the quantity of resource available which may influence group sizes, that is, the presence of small prides may favor individualistic foraging while large prides may form intermediate‐sized groups when prey is scarce (Mosser & Packer, 2009).

Our results also revealed significant differences between study sites, with Mara having a significantly larger lion group size compared to Tsavo, Laikipia and Amboseli. The Mara is characterized by higher densities and diversities of large mammals (John Waweru et al., 2021), and large lion group sizes have often been linked to high prey biomass (McEvoy et al., 2022). In Amboseli, despite the success of community conservation efforts in reducing conflicts in the GR leading to an expanding lion population, the smaller group sizes could be attributed to a history of persecution that may lead to the observed smaller groups (Dolrenry, 2013). Additionally, the Amboseli region has, currently and historically, had lower densities of lions than the Mara region.

We collected our data across all study sites primarily during the dry season in Kenya. Therefore, our findings reflect lion grouping patterns during this season. Climate variability causes changes in prey availability and therefore in lion group size, for example, in 2009 after an extreme drought period in Amboseli National Park, the mean lion group size reduced from an average of 3.5 to 1.35 (Tuqa et al., 2014). We note that prey abundance and distribution is also likely to be an important variable, but we did not have comparable data.

In line with our hypothesis, the relationship between lion subgroup size and the non‐tree cover in Amboseli and Tsavo showed an optimal pattern, where larger lion subgroups were associated with a moderate proportion of non‐tree cover. While in Samburu the non‐tree cover exhibited a linear relationship with lion subgroup size and it increased with an increase in the proportion of non‐tree cover. Non‐tree cover provides important ambush opportunities and may also serve as a refuge from potential encounters with people and livestock (Mosser & Packer, 2009; Oriol‐Cotterill et al., 2015). This may be particularly important in the Amboseli and Samburu study sites due to the presence of human settlements and livestock within these areas (Bhalla, 2017; Dolrenry, 2013), and the frequent illegal livestock incursion into Tsavo (John Waweru et al., 2021).

Also in line with our hypothesis, lion subgroup size decreased with increased distance from water in Laikipia, Mara, Meru, Samburu, and Nakuru. We attribute this to the study being carried out during the dry season in these study sites when herbivores are known to aggregate around water sources, thus forming important habitat for lions that are vital for reproduction and hunting success (Hopcraft et al., 2005; Mosser & Packer, 2009; Valeix et al., 2010). Therefore, maintaining access to such limited resources will have positive fitness outcomes, and this can be achieved by forming large groups closer to resources as larger groups have a higher probability of prevailing in disputes over territory (Valeix et al., 2012). However, in Nairobi, the results were contrary to our hypothesis, and the lion subgroup size increased with increased distance from rivers. We can presume this was the case since the survey period in Nairobi also covered the wet season hence wild herbivores did not need to aggregate close to water. We also note that four of our sites (Meru, Samburu, Nairobi, and Nakuru) contain relatively small populations with fewer groups, and some caution is warranted when interpreting the results from these sites. These sites exhibited notably low marginal and conditional *R* ^2^ measures, possibly indicating that their models explained little variation in subgroup size. Considering our analysis used four variables to assess their influence on lion subgroup size, inclusion of additional predictor variables, such as prey abundance, may offer further insights for these sites.

We note that a limitation of our study is that it was conducted over a relatively short period of time, meaning that we did not acquire in‐depth knowledge of the social groups. This meant it was difficult for us to resolve whether our results reflect variation in group size itself, or the manifestations of fission fusion dynamics. For example, that lions were found in smaller groups closer to human settlements could be because these groups have been persecuted and are diminished in number, or it could be because they break into smaller groups when close to settlements. While this nuanced understanding may evade our study, our multi‐site approach provides insights at a national scale and regardless of whether our results reflect absolute group size or fission fusion dynamics, our results suggest that at a national level, lion grouping patterns are affected by key anthropogenic and ecological variables. In light of our results, we suggest that (1) regular monitoring of lion populations is conducted to build upon the knowledge base we have created; (2) managers should strive to maintain suitable habitats that provide cover for hunting, the protection of cubs, and refuge from humans; (3) water sources should be protected and wherever possible, free from human disturbance; (4) anthropogenic activities close to lion habitats should be minimized. Finally, in line with our results and the national recovery and management plan for lions in Kenya 2020–2030 we recommend the development of a site‐specific approach to lion conservation. This should involve the establishment of collaborative landscape‐level lion conservation units, encompassing government‐protected areas, community conservancies, private conservation areas, and group ranches.

## AUTHOR CONTRIBUTIONS

**Mumbi Chege:** Conceptualization (equal); data curation (equal); formal analysis (equal); funding acquisition (equal); investigation (equal); methodology (equal); validation (equal); writing – original draft (lead); writing – review and editing (equal). **Laura D. Bertola:** Data curation (equal); supervision (equal); writing – review and editing (equal). **Geert R. De Snoo:** Supervision (equal); writing – review and editing (equal). **Shadrack Ngene:** Writing – review and editing (equal). **Tobias Otieno:** Investigation (equal); writing – review and editing (equal). **Irene Amoke:** Funding acquisition (equal); writing – review and editing (equal). **Maarten van 't Zelfde:** Data curation (equal). **Stephanie Dolrenry:** Funding acquisition (equal); investigation (equal); writing – review and editing (equal). **Femke Broekhuis:** Conceptualization (equal); funding acquisition (equal); investigation (equal); writing – review and editing (equal). **Will Tamis:** Formal analysis (equal); methodology (equal); validation (equal). **Hans H. De Iongh:** Conceptualization (equal); methodology (supporting); supervision (equal); writing – review and editing (equal). **Nicholas B. Elliot:** Conceptualization (equal); data curation (equal); formal analysis (equal); funding acquisition (equal); investigation (equal); methodology (equal); supervision (equal); validation (equal); writing – review and editing (equal).

## CONFLICT OF INTEREST STATEMENT

The authors declare no conflicts of interest.

## Data Availability

Restrictions apply to the availability of these data, which were used under license for this study. We uploaded a section of the data that support the findings of this study as supporting material for review purposes.

## APPENDIX 1

### Model outputs

**TABLE A1:** Model outputs tables showing the significant variables for all sites, Mara, Tsavo, Amboseli, and Laikipia (*Significant codes and definition of Incidence Rate ratios indicated below table and confidence Intervals are shown in brackets in the table).

Predictors	All sites		Mara		Tsavo		Amboseli		Laikipia	
	Incidence rate ratios	*p*	Incidence rate ratios	*p*	Incidence rate ratios	*p*	Incidence rate ratios	*p*	Incidence rate ratios	*p*
Distance to a boundary	0.87* (0.76–1.00)	**.044**	0.76** (0.63–0.92)	**.004**					0.80 (0.62–1.02)	.070
Non‐tree vegetation	1.14*** (1.06–1.23)	**.001**			1.20* (1.01–1.42)	**.036**	1.26* (1.03–1.53)	**.023**		
I(Non‐tree^2^)	1.00*** (1.00–1.00)	**.001**			1.00* (1.00–1.00)	**.012**	1.00* (1.00–1.00)	**.034**		
Distance to rivers	0.89* (0.81–0.97)	**.012**	0.83* (0.70–0.99)	**.034**					0.85* (0.72–1.00)	**.049**
Distance to human settlements	1.14* (1.01–1.30)	**.040**							1.53** (1.18–1.98)	**.001**
Random effects										
*σ* ^2^	0.65		0.79		0.24		0.70		0.80	
*τ*_00_	0.45_groupID_		0.28_groupID_		0.57_groupID_		0.32_groupID_		0.53_groupID_	
	0.41_ID_		0.58_ID_				0.42_ID_		0.55_ID_	
ICC	0.41		0.26		0.71		0.31		0.40	
*N*	199_groupID_		59_groupID_		42_groupID_		30_groupID_		54_groupID_	
	798_ID_		221_ID_				95_ID_		231_ID_	
Observations	798		221		115		95		231	
Marginal *R* ^2^/Conditional *R* ^2^	.056/.440		.108/.340		.094/.734		.106/.386		.068/.438	

*Note*: Incidence rate ratio >1 indicates positive relationship, <1 indicates negative relationship and =1 indicates no relationship with variable. Bold values indicate the significant variables.

**p* < .05; ***p* < .01; ****p* < .001.

**TABLE A2:** Model outputs tables showing the significant variables for Meru, Nairobi, Nakuru, and Samburu (*Significant codes and definition of Incidence Rate Ratios indicated below table and Confidence Intervals are shown in brackets in the table).

Predictors	Meru		Nairobi		Nakuru		Samburu	
	Incidence rate ratios	*p*	Incidence rate ratios	*p*	Incidence rate ratios	*p*	Incidence rate ratios	*p*
Distance to a boundary	0.65* (0.46–0.92)	**.015**			0.25*** (0.15–0.41)	**<.001**		
Distance to rivers	0.81 (0.63–1.04)	.094	1.39 (0.82–2.35)	.220	0.68* (0.49–0.93)	**.015**	0.70** (0.53–0.91)	**.009**
Distance to human settlements					3.78*** (2.05–6.95)	**<.001**	0.78 (0.60–1.00)	.052
Non‐tree vegetation							1.01* (1.00–1.02)	**.035**
Random effects								
*σ* ^2^	0.23		1.10		0.34		0.23	
*τ*_00_	0.00_groupID_		0.00_groupID_		0.00_groupID_		0.00_groupID_	
			0.75_ID_					
ICC	0.00							
*N*	5_groupID_		2_groupID_		2_groupID_		5_groupID_	
			23_ID_					
Observations	39		23		33		41	
Marginal *R* ^2^/Conditional *R* ^2^	.496/.497		.090/NA		.606/NA		.343/NA	

*Note*: Incidence rate ratio >1 indicates positive relationship, <1 indicates negative relationship, and =1 indicates no relationship with variable. Bold values indicate the significant variables.

**p* < .05; ***p* < .01; ****p* < .001.

## References

[ece310982-bib-0001] Bhalla, S. (2017). Demography and ranging behaviour of lions (Panthera leo) within a human‐occupied landscape in northern Kenya . Retrieved from https://ewasolions.org/wp‐content/uploads/2020/09/Shivani‐Bhalla‐Thesis‐2017‐compressed.pdf

[ece310982-bib-0002] Borrego, N. , Ozgul, A. , Slotow, R. , & Packer, C. (2018). Lion population dynamics: Do nomadic males matter? Behavioral Ecology, 29(3), 660–666. 10.1093/beheco/ary018

[ece310982-bib-0003] Broekhuis, F. , Ngene, S. , Gopalaswamy, A. M. , Mwaura, A. , Dloniak, S. M. , Ngatia, D. K. , Tyrrell, P. D. , Yamane, Y. , & Elliot, N. B. (2022). Predicting potential distributions of large carnivores in Kenya: An occupancy study to guide conservation. Diversity and Distributions, 28(7), 1445–1457. 10.1111/ddi.13554

[ece310982-bib-0005] Courchamp, F. , Rasmussen, G. S. A. , & Macdonald, D. W. (2002). Small pack size imposes a trade‐off between hunting and pup‐guarding in the painted hunting dog Lycaon pictus. Behavioral Ecology, 13(1), 20–27. 10.1093/beheco/13.1.20

[ece310982-bib-0006] Denboba, M. A. (2022). Grazing management and carbon sequestration in the Dry Lowland Rangelands of Southern Ethiopia. Sustainable Environment, 8(1), 2046959. 10.1080/27658511.2022.2046959

[ece310982-bib-0007] Dickman, A. J. , Macdonald, E. A. , & Macdonald, D. W. (2011). A review of financial instruments to pay for predator conservation and encourage human‐carnivore coexistence. Proceedings of the National Academy of Sciences of the United States of America, 108(34), 13937–13944. 10.1073/pnas.1012972108 21873181 PMC3161573

[ece310982-bib-0008] Dolrenry, S. (2013). *African lion (Panthera leo) behaviour, monitoring, and survival in human‐dominated landscapes* (PhD thesis). University of Wisconsin‐Madison.

[ece310982-bib-0009] Elliot, N. B. , Broekhuis, F. , Omondi, P. , Ngene, S. , Kariuki, L. , Sankan, K. , Chege, M. , Wato, Y. , Amoke, I. , Dolrenry, S. , & Gopalaswamy, A. M. (2021). Report on the application of novel estimating methodologies to monitor lion abundance within source populations and large carnivore occupancy at a national scale .

[ece310982-bib-0010] Elliot, N. B. , & Gopalaswamy, A. M. (2017). Toward accurate and precise estimates of lion density. Conservation Biology: The Journal of the Society for Conservation Biology, 31(4), 934–943. 10.1111/cobi.12878 27958641

[ece310982-bib-0011] Gager, Y. , Gimenez, O. , O'Mara, M. T. , & Dechmann, D. K. N. (2016). Group size, survival and surprisingly short lifespan in socially foraging bats. BMC Ecology, 16(1), 1–12. 10.1186/s12898-016-0056-1 26767616 PMC4714502

[ece310982-bib-0013] Harcourt, A. H. , Parks, S. A. , & Woodroffe, R. (2001). Human density as an influence on species/area relationships: Double jeopardy for small African reserves? Biodiversity and Conservation, 10, 1011–1026.

[ece310982-bib-0012] Hopcraft, J. G. C. , Sinclair, A. R. E. , & Packer, C. (2005). Planning for success: Serengeti lions seek prey accessibility rather than abundance. Journal of Animal Ecology, 74(3), 559–566. 10.1111/j.1365-2656.2005.00955.x

[ece310982-bib-0014] IGAD . (2010). Land Governance in IGAD Region ‐ Assessment of land governance framework, training & research land and governance institutions_Kenya Country Profile . 10.1787/9789264085886-2-en

[ece310982-bib-0015] John Waweru, B. , Omondi, P. , Ngene, S. , Mukeka, J. , Wanyonyi, E. , Ngoru, B. , Mwiu, S. , Muteti, D. , Lala, F. , Kariuki, L. , Ihwagi, F. , Kiambi, S. , Khyale, C. , Bundotich, G. , Omengo, F. , Hongo, P. , Maina, P. , Muchiri, F. , Omar, M. , & Kanga, E. (2021). National wildlife census 2021 report_abridged version . Retrieved from www.digimatt.co.ke

[ece310982-bib-0016] Kenya Wildlife Conservancies Association . (2019). Strategic plan 2019–2023 . Retrieved from https://kwcakenya.com/

[ece310982-bib-0017] Kenya Wildlife Service . (2016). Conservation and management strategy for lion and spotted hyena in Kenya_2009–2014. Kenya Wildlife Service.

[ece310982-bib-0018] Kenya Wildlife Service . (2020). National recovery and action plan for lion and spotted hyena in Kenya (2020–2030) national recovery and action plan for lion and spotted hyena in Kenya (2nd ed.). Kenya Wildlife Service.

[ece310982-bib-0019] Krause, J. , & Ruxton, G. (2002). Living in groups. Oxford series in ecology and evolution. Oxford University Press.

[ece310982-bib-0020] Kuznetsova, A. , Brockhoff, P. B. , & Christensen, R. H. B. (2017). lmerTest package: Tests in linear mixed effects models. Journal of Statistical Software, 82(13), 1–26. 10.18637/JSS.V082.I13

[ece310982-bib-0021] Lesilau, F. , Verschueren, S. , Zelfde, M. V. , Musters, K. C. J. M. , Snoo, G. R. D. , & Iongh, H. H. D. (2021). Spatial ecology of lions in a small, semi‐fenced park surrounded by dense human populations: The case study of Nairobi National Park, Kenya. Mammalia, 85(3), 198–207. 10.1515/mammalia-2020-0116

[ece310982-bib-0022] Loveridge, A. J. , Hemson, G. , Davidson, Z. , & Macdonald, D. W. (2010). African lions on the edge: reserve boundaries as ‘attractive sinks’. Biology and Conservation of Wild Felids, 2021, 283–304.

[ece310982-bib-0023] Majolo, B. , & Huang, P. (2018). Group living. In J. Vonk & T. Shackelford (Eds.), Encyclopedia of animal cognition and behavior (pp. 1–12). Springer International Publishing. 10.1007/978-3-319-47829-6_1865-1

[ece310982-bib-0024] Markham, A. C. , Gesquiere, L. R. , Alberts, S. C. , & Altmann, J. (2015). Optimal group size in a highly social mammal. Proceedings of the National Academy of Sciences of the United States of America, 112, 14882–14887. 10.5061/dryad.nh597 26504236 PMC4672815

[ece310982-bib-0025] Mbizah, M. M. , Farine, D. R. , Valeix, M. , Hunt, J. E. , Macdonald, D. W. , & Loveridge, A. J. (2020). Effect of ecological factors on fine‐scale patterns of social structure in African lions. Journal of Animal Ecology, 89(11), 2665–2676. 10.1111/1365-2656.13334 32895921

[ece310982-bib-0026] McEvoy, O. K. , Ferreira, S. M. , & Parker, D. M. (2022). The impacts of management interventions on the sociality of African lions (*Panthera leo*): Implications for lion conservation. Ecological Solutions and Evidence, 3(2), e12135. 10.1002/2688-8319.12135

[ece310982-bib-0027] Miller, J. R. B. , Balme, G. , Lindsey, P. A. , Loveridge, A. J. , Becker, M. S. , Begg, C. , Brink, H. , Dolrenry, S. , Hunt, J. E. , Jansson, I. , Macdonald, D. W. , Mandisodza‐Chikerema, R. L. , Cotterill, A. O. , Packer, C. , Rosengren, D. , Stratford, K. , Trinkel, M. , White, P. A. , Winterbach, C. , & Funston, P. J. (2016). Aging traits and sustainable trophy hunting of African lions. Biological Conservation, 201, 160–168. 10.1016/j.biocon.2016.07.003

[ece310982-bib-0028] Mosser, A. , Fryxell, J. M. , Eberly, L. , & Packer, C. (2009). Serengeti real estate: Density vs. fitness‐based indicators of lion habitat quality. Ecology Letters, 12(10), 1050–1060. 10.1111/j.1461-0248.2009.01359.x 19708970

[ece310982-bib-0029] Mosser, A. , & Packer, C. (2009). Group territoriality and the benefits of sociality in the African lion, *Panthera leo* . Animal Behaviour, 78(2), 359–370. 10.1016/j.anbehav.2009.04.024

[ece310982-bib-0030] Mosser, A. A. , Kosmala, M. , & Packer, C. (2015). Landscape heterogeneity and behavioral traits drive the evolution of lion group territoriality. Behavioral Ecology, 26(4), 1051–1059. 10.1093/beheco/arv046

[ece310982-bib-0031] Mudumba, T. , Omoya, E. , Nsubuga, J. , & Plumptre, A. (2015). Home ranges of Ishasha lions: Size and location in relation to habitat and prey availability. Journal of East African Natural History, 104, 227–246. 10.2982/028.104.0115

[ece310982-bib-0032] Mukeka, J. M. , Ogutu, J. O. , Kanga, E. , & Røskaft, E. (2020). Spatial and temporal dynamics of human – Wildlife conflicts in the Kenya Greater Tsavo. Ecosystems, 14(2), 255–272.

[ece310982-bib-0033] Ogutu, J. O. , Piepho, H. P. , Said, M. Y. , Ojwang, G. O. , Njino, L. W. , Kifugo, S. C. , & Wargute, P. W. (2016). Extreme wildlife declines and concurrent increase in livestock numbers in Kenya: What are the causes? PLoS One, 11(9), e0163249. 10.1371/journal.pone.0163249 27676077 PMC5039022

[ece310982-bib-0034] Okello, M. M. , Kenana, L. , Maliti, H. , Kiringe, J. W. , Kanga, E. , Warinwa, F. , Bakari, S. , Gichohi, N. , Ndambuki, S. , Kija, H. , Sitati, N. , Kimutai, D. , Mwita, M. , Muteti, D. , & Muruthi, P. (2015). Population status and trend of water dependent grazers (buffalo and waterbuck) in the Kenya‐Tanzania borderland. Natural Resources, 6, 91–114. 10.4236/nr.2015.62009

[ece310982-bib-0035] Oriol‐Cotterill, A. , Macdonald, D. W. , Valeix, M. , Ekwanga, S. , & Frank, L. G. (2015). Spatiotemporal patterns of lion space use in a human‐dominated landscape. Animal Behaviour, 101, 27–39. 10.1016/j.anbehav.2014.11.020

[ece310982-bib-0036] Packer, C. , Scheel, D. , & Pusey, A. E. (1990). Why lions form groups: Food is not enough. American Naturalist, 136(1), 1–19. 10.1086/285079

[ece310982-bib-0037] Pennycuick, C. J. , & Rudnai, J. (1970). A method of identifying individual lions (*Panthera leo*), with an analysis of reliability of identification. Journal of Zoology, 160(4), 497–508.

[ece310982-bib-0038] R Core Team . (2018). R: A language and environment for statistical computing. R Foundation for Statistical Computing. Retrieved from https://www.r‐project.org

[ece310982-bib-0039] Schaller, G. B. (1972). The Serengeti lion: A study of predator‐prey relations. University of Chicago Press. Retrieved from https://books.google.mw/books?id=rZP1MIJWiKsC

[ece310982-bib-0040] Smuts, G. L. , Hanks, J. , & Whyte, J. (1978). Reproduction and social organisation of lions from the Kruger National Park. Carnivore, 1, 17–28.

[ece310982-bib-0041] Spong, G. (2002). Space use in lions, Panthera leo, in the Selous Game Reserve: social and ecological factors. Behavioral Ecology and Sociobiology, 52, 303–307. 10.1007/s00265-002-0515-x

[ece310982-bib-0042] The Wildlife Conservation and Management ACT . (2013). GoK Nairobi 116 .

[ece310982-bib-0043] Thuo, D. N. , Kamau, J. , Amimo, J. O. , & Kibegwa, F. (2015). Population viability analysis of black rhinoceros (*Diceros bicornis michaeli*) in Lake Nakuru National Park, Kenya. Journal of Biodiversity & Endangered Species, 3(1), 1–5. 10.4172/2332-2543.1000150

[ece310982-bib-0044] Tuqa, J. H. , Funston, P. , Musyoki, C. , Ojwang, G. O. , Gichuki, N. N. , Bauer, H. , hans, Tamis, W. , Dolrenry, S. , Van't Zelfde, M. , de Snoo, G. R. , & de Iongh, H. H. (2014). Impact of severe climate variability on lion home range and movement patterns in the Amboseli ecosystem, Kenya. Global Ecology and Conservation, 2, 1–10. 10.1016/j.gecco.2014.07.006

[ece310982-bib-0045] Valeix, M. , Loveridge, A. J. , Davidson, Z. , Madzikanda, H. , Fritz, H. , & Macdonald, D. W. (2010). How key habitat features influence large terrestrial carnivore movements: Waterholes and African lions in a semi‐arid savanna of north‐western Zimbabwe. Landscape Ecology, 25(3), 337–351. 10.1007/s10980-009-9425-x

[ece310982-bib-0046] Valeix, M. , Loveridge, A. J. , & Macdonald, D. W. (2012). Influence of prey dispersion on territory and group size of African lions: A test of the resource dispersion hypothesis. Ecology, 93(11), 2490–2496. 23236920 10.1890/12-0018.1

[ece310982-bib-0047] VanderWaal, K. L. , Mosser, A. , & Packer, C. (2009). Optimal group size, dispersal decisions and postdispersal relationships in female African lions. Animal Behaviour, 77(4), 949–954. 10.1016/j.anbehav.2008.12.028

[ece310982-bib-0048] Woodroffe, R. , & Ginsberg, J. R. (1998). Edge effects and the extinction of populations inside protected areas. Science, 280(5372), 2126–2128. 10.1126/science.280.5372.2126 9641920

[ece310982-bib-0049] Woodroffe . (2000). Predators and people: Using human densities to interpret declines of large carnivores. Animal Conservation, 3, 165–173. 10.1111/j.1469-1795.2000.tb00241.x

[ece310982-bib-0050] Zhou, Y. , Liu, B. , Mbuni, Y. , Yan, X. , Mwachala, G. , Hu, G. , & Wang, Q. (2017). Vascular flora of Kenya, based on the flora of tropical East Africa. PhytoKeys, 90, 113–126. 10.3897/phytokeys.90.20531 PMC5740400 29308037

